# Supplementary material for: A Systematic Review and Meta-Analysis of Supramarginal Resection versus Gross Total Resection in Glioblastoma: Can We Enhance Progression-Free Survival Time and Preserve Postoperative Safety?
Source: Cancers (Basel). 2023 Mar 15;15(6):1772. doi: 10.3390/cancers15061772 (PMC10046815; doi:10.3390/cancers15061772)
Supplement: Supplementary file 1 [file cancers-15-01772-s001.zip › cancers-2280145-supplementary.pdf]

Supplementary Table S1. Postoperative Karnofsky performance status and new neurological deficits.

Study	Postoperative KPS	New Neurological Deficits
Glenn et al., 2018 [24]	NA	SMR: 1/7 (dysphasia in one patient)<br>GTR: 0/9
De Bonis et al., 2013 [25]	Mean (range): 80 (50–100) (not stratified by EOR)	NA
Schneider et al., 2019 [26]	12-month follow-up:<br>SMR: KPS ≥ 70: 11/14<br>GTR: KPS ≥ 70: 10/24	NA
Pessina et al., [27] 2017	NA	SMR: 0/21<br>GTR: 0/60
Roh et al., [28] 2019	Median (range):<br>SMR: 80 (40–100)<br>GTR: 80 (40–100)	NA
Shah et al., [29] 2020	Median (IQR):<br>SMR: 80 (60–90)<br>GTR: 80 (70–90)	NA
Mampre et al., [30] 2018	NA	Mean percent of FLAIR resection<br>New deficits: 29.57%<br>No new deficits: 25.38%
Hamada et al., [31] 2016	NA	9/59 motor deficits (not stratified by EOR)
Li et al., [32] 2016	NA	Deficits of FLAIR-guided SMR dichotomized into <53.21% (n = 159)/≥ 53.21% (n = 484) FLAIR resections:<br>Motor deficits: 8/159 and 49/484<br>Speech impairment: 9/159 and 37/484<br>Visual impairment: 4/159 and 14/484<br>Sensory deficit:1/159 and 1/484<br>Cranial nerve deficit: 1/159 & 3/484<br>Cognitive/memory/mental problems: 3/159 and 5/484 (deficits not compared with conventional GTR)
Schneider et al., [33] 2020	At discharge:<br>SMR: 15/20<br>GTR: 29/41	Only language and visual deficits described:<br>SMR: 0/20<br>GTR: 0/41
Figueroa et al., [34] 2020	Mean value: 83 (not stratified by EOR)	Speech impairment: 2/57<br>Visual impairment: 1/57

		Cranial nerve deficit: 1/57<br>Psychosis: 1/57<br>Sensory: 1/57<br>Mood disorder: 1/57<br>(deficits not stratified by EOR)

Abbreviation: EOR = extent of resection; FLAIR = fluid-attenuated inversion recovery; GTR = gross total resection; IQR = interquartile range; KPS = Karnofsky performance status; NA = not available; SMR = supramarginal resection.

Section and Topic	Item #	Checklist item	Location where item is reported
**TITLE**			
Title	1	A Systematic Review and Meta-Analysis of Supramarginal Resection versus Gross Total Resection in Glioblastoma: Can We Enhance Progression-Free Survival Time and Preserve Postoperative Safety?	Title
**ABSTRACT**			
Abstract	2	To date, gross total resection (GTR) of the contrast-enhancing area of glioblastoma (GB) is the benchmark treatment regarding surgical therapy. However, GB infiltrates beyond those margins, and most tumors recur in close proximity to the initial resection margin. It is unclear whether a supramarginal resection (SMR) enhances progression-free survival (PFS) time without increasing the incidence of postoperative surgical complications. The aim of the present meta-analysis was to investigate SMR with regard to PFS and postoperative surgical complications. We searched for eligible studies comparing SMR techniques with conventional GTR in PubMed, Cochrane Library, Web of Science, and Medline databases. From 3158 initially identified records, 11 articles met the criteria and were included in our meta-analysis.	Abstract
**INTRODUCTION**			
Rationale	3	While aggressive surgical approaches are essential regarding tumor control, preserving neurological function and avoiding complications should remain the second priority of surgery [14]. A recent meta-analysis found a moderate OS benefit for patients who underwent a supramarginal resection (SMR) [15]. Tumor progression is a hallmark in GB because recurrent GBs are driven by intrinsic (e.g., MGMT upregulation and increased tumor mutation burden) and extrinsic (e.g., hypoxia and immuno-suppressive tumor microenvironments) mechanisms developing resistance to therapies [16]. Furthermore, GB progression and postoperative complications are also significantly associated with negative changes in patient-reported health-related quality of life [17,18]. To date, the impact of supramarginal resection (SMR) compared to gross total resection (GTR) on progression-free survival and postoperative complications (e.g., mortality, meningitis, intracranial hemorrhage, and cerebrospinal fluid (CSF) leaks) has not yet been investigated in a meta-analysis.	Introduction
Objectives	4	The present systematic review and meta-analysis aim to investigate supramarginal resection compared with gross total resection regarding the probability of progression-free survival and perioperative surgical complications.	Introduction
**METHODS**			
Eligibility criteria	5	The authors performed a systematic search in November, 2022 of the PubMed, Cochrane Library, Web of Science, and Medline databases using the search terms “glioblastoma”, “supratotal resection”, “supramaximal resection”, “supracomplete resection”, “FLAIR resection”, “lobectomy”, and “supramarginal resection”. The search was limited to “human studies”, “clinical trials”, and “English” language publications, with a literature search that included all results up to 31 October 2022. The inclusion criteria were formulated using the PICOS (population, intervention, comparator, outcomes, and study design) framework [21], with the following criteria: patients had undergone treatment for GB; relevant surgical resections were performed; SMR results were compared to conventional GTR regarding PFS or perioperative complications; all prespecified endpoints were reported; and the studies were comparative studies comparing different surgical resection techniques. Records such as reviews, study protocols, letters, conference abstracts, unpublished papers, animal experiments, and studies with insufficient data (e.g., no description of surgical resection technique with definition of extent of resection) were excluded. Previous meta-analyses and reviews were also searched for studies matching the inclusion and exclusion criteria.	Methods
Information sources	6	We searched the PubMed, Cochrane Library, Web of Science, and Medline databases using the search terms “glioblastoma”, “supratotal resection”, “supramaximal resection”, “supracomplete resection”, “FLAIR resection”, “lobectomy”, and “supramarginal resection”.	Methods
Search strategy	7	The search was limited to “human studies”, “clinical trials”, and “English” language publications, with a literature search that included all results up to 31 October 2022. The inclusion criteria were formulated using the PICOS (population, intervention, comparator, outcomes, and study design) framework [21], with the following criteria: patients had undergone treatment for GB; relevant surgical resections were performed; SMR	Methods

Section and Topic	Item #	Checklist item	Location where item is reported
		results were compared to conventional GTR regarding PFS or perioperative complications; all prespecified endpoints were reported; and the studies were comparative studies comparing different surgical resection techniques.	
Selection process	8	The identified articles were further examined in a stepwise workflow that involved screening titles of the studies, abstracts, and full texts.	Methods
Data collection process	9	The data collection was performed by two authors independently (MV, JW). The disagreement between both reviewers was settled by third author (EG)	Methods
Data items	10a	We analyzed the studies to conduct a meta-analysis according to following outcomes:<br>1) PFS times stratified by GTR and SMR<br>2) Multivariate Cox regression analysis of PFS in studies comparing GTR with SMR<br>3) Postoperative CSF leaks in studies comparing GTR with SMR<br>4) Postoperative intracranial hemorrhage in studies comparing GTR with SMR<br>5) Postoperative meningitis in studies comparing GTR with SMR<br>6) Mortality in in studies comparing GTR with SMR<br>7) Postoperative KPS in studies comparing GTR with SMR<br>8) Postoperative neurological deficits in studies comparing GTR with SMR	Methods
	10b	- PFS was defined as the time from initial treatment to tumor progression<br>- CSF leaks, intracranial hemorrhage, and meningitis were defined as complications necessitating medical treatment<br>- Mortality was assessed according to provided data as patients, who deceased in the course of the therapy	Methods
Study risk of bias assessment	11	To investigate the statistical heterogeneity and inconsistency, x^2 and I^2 statistics were used respectively; an I^2 value of 50% or more represented substantial heterogeneity. Weight to the size of each study was involved with regard of the estimation of treatment effects. Funnel plots were used to examine the publication bias of included studies. Begg´s test was used to statistically analyze publication bias. Effect sizes were expressed as pooled OR estimates.	Methods
Effect measures	12	We measured and reported outcomes in Forest-plots, providing heterogeneity and inconsistency analysis, pooled odds ratio and statistical significance.	Methods
Synthesis methods	13a	To see all the eligible studies and reported outcomes, see Table 1	Methods
	13b	To see all the eligible studies and reported outcomes, see Table 1	Methods
	13c	For visualisation of our meta-analysis, Forest Plots and Funnel Plots were created with Review Manager Web (RevMan Web Version 5.4.1 from the Cochrane Collaboration, available at revman.cochrane.org) and were presented as figures	Methods
	13d	The “Generic inverse variance” method was used for statistical analysis, whereby a pooled hazard ratio (HR) was determined from the natural logarithm (LN) of the individual HR (LN (HR)) and the corresponding 95% confidence interval (CI). For the hazard ratio (HR), the standard error (SE) for the LN (OR) was calculated from the 95% CI using the following formula: SE = (LN (upper CI limit) − LN (lower CI limit))/3.92 (according to the *Cochrane Handbook for Systematic Reviews of Interventions* version 6.3 [20]). Standard deviations were obtained from the 95% CI limits. Statistical heterogeneity and inconsistency were investigated using x^2 and I^2 statistics, respectively, where an I^2 value of 50% or more indicated substantial heterogeneity [22]. Begg´s tests were conducted using MedCalc (version 20.123 for Windows with a *p*-value <0.05 considered indicative of bias. Pooled OR and pooled HR estimates were used to express the effect sizes, and the following endpoints were investigated: progression-free survival, postoperative meningitis, postoperative intracranial hemorrhage, and postoperative CSF leaks Effect sizes were expressed as pooled OR estimates.	Methods

Section and Topic	Item #	Checklist item	Location where item is reported
	13e	In case of subgroup-analysis, there might be a heterogeneity of the data caused by lack of published results	Methods
	13f	Not available	Methods
Reporting bias assessment	14	According to our strict inclusion criteria of included studies, we do not suppose to have missing results	Methods
Certainty assessment	15	Not available	Methods
**RESULTS**			
Study selection	16a	Process of the search and selection is summarized in Figure 1	Figure 1
	16b	Not available	NA
Study characteristics	17	Table 1, Table 2 & Table 3	Tables 1-3
Risk of bias in studies	18	Not available	NA
Results of individual studies	19	Analysis on the defined outcomes is separately reported on with its own forest plots	Figures 2-6
Results of syntheses	20a	Publication bias is summarized in funnel-plots in each analysis separately	Figures 7&8
	20b	Presented in sections 3.3-3.6	Sections 3.3-3.6
	20c	Presented in sections 3.3-3.6	Sections 3.3-3.6
	20d	Presented in sections 3.3-3.6	Sections 3.3-3.6
Reporting biases	21	Not available	NA
Certainty of evidence	22	Not available	NA
**DISCUSSION**			
Discussion	23a	Provide a general interpretation of the results in the context of other evidence.	Discussion
	23b	Discuss any limitations of the evidence included in the review.	Discussion, last paragraph
	23c	Discuss any limitations of the review processes used.	NA due to strict selection criteria
	23d	Discuss implications of the results for practice, policy, and future research.	Discussion

Section and Topic	Item #	Checklist item	Location where item is reported
**OTHER INFORMATION**			
Registration and protocol	24a	PROSPERO Registration: CRD42023395933	Methods
	24b	PROSPERO Registration: CRD42023395933	Methods
	24c	No amendments to describe	
Support	25	There is no financial support to describe	Manuscript
Competing interests	26	No interest to declare	Manuscript
Availability of data, code and other materials	27	Template data collection form extracted data and used data for the analysis are reported in the section methods and results. The softwares which have been used to conduct the meta-analysis are available online.	Methods, Results

From: Page MJ, McKenzie JE, Bossuyt PM, Boutron I, Hoffmann TC, Mulrow CD, et al. The PRISMA 2020 statement: an updated guideline for reporting systematic reviews. BMJ 2021;372:n71. doi: 10.1136/bmj.n71
For more information, visit: http://www.prisma-statement.org/

Supplementary Figure S1. PRISMA checklist of the present meta-analysis [19].
